# High-Performance Terahertz Detection via Quasi-2D Perovskite/Weyl Semimetal Heterojunction

**DOI:** 10.3390/ma19091847

**Published:** 2026-04-30

**Authors:** Chao Feng, Baoxing Liu, Haoyi Ning, Leying Hua, Zhixiang Zheng, Shuhong Li, Wenjun Wang, Yunlong Liu

**Affiliations:** School of Physical Science and Information Technology, Liaocheng University, Liaocheng 252059, Chinawjwang@lcu.edu.cn (W.W.)

**Keywords:** perovskite, Weyl semimetal, heterojunction, terahertz detector

## Abstract

**Highlights:**

- A novel quasi-2D perovskite/Weyl semimetal (Co_3_Sn_2_S_2_) heterojunction is fabricated for THz detection.
- The detector achieves a record-high responsivity of 374.15 A/W and an *NEP* of 0.29 pW·Hz^−1/2^ at 0.1 THz.
- Band engineering and 455 nm laser treatment synergistically enhance THz absorption and carrier extraction.

**Abstract:**

Terahertz radiation exhibits significant potential for communications, imaging, and spectroscopy. However, the development of efficient and low-cost THz detectors remains challenging due to limitations such as insufficient sensitivity, slow response speed, and poor room temperature stability. This work presents an innovative quasi-2D perovskite/Weyl semimetal (Co_3_Sn_2_S_2_) heterojunction THz detector that combines complementary material properties via band engineering. The device achieves a remarkable responsivity of 374.15 A/W, a specific detectivity of 6.27 × 10^11^ cm·Hz^1/2^·W^−1^, and a noise-equivalent power of 0.29 pW·Hz^−1/2^ at 0.1 THz. This performance stems from the strong THz absorption of the perovskite layer combined with the high carrier mobility and topological surface states of the Co_3_Sn_2_S_2_, which collectively enable ultrafast carrier extraction and suppressed interfacial recombination. This heterojunction design offers a novel strategy for high-performance terahertz detection and facilitates its integration into next-generation portable, integrated devices.

## 1. Introduction

Terahertz technology (0.1–10 THz) has shown significant application prospects in 6G mobile communication [1], security detection [2], and biomedical imaging [3] due to its unique non-ionizing and penetrating capabilities. Terahertz components have long been used in filters [4], optical absorbers [5], and modulators [6]. However, the translation of terahertz technology into practical applications is hindered by limitations in core detection devices. Efficient terahertz detectors are essential and must meet three key requirements: high quantum efficiency, room temperature operation, and low-cost fabrication [7]. For point-of-care or wearable applications, they must operate stably at room temperature with low power consumption and be compatible with cost-effective, large-scale manufacturing. Currently, mainstream technological approaches possess inherent, difficult-to-overcome shortcomings that prevent them from meeting these stringent practical requirements. Conventional group-IV semiconductors (Ge, Si) exhibit terahertz quantum efficiencies below 0.1%, mainly due to the severe mismatch between the interband transition energy and the terahertz photon energy [8]. Detectors based on superconducting materials can achieve nearly saturated detection efficiency and high sensitivity, but they require an extreme low-temperature environment below 4.2 K [9], which significantly increases the mass, volume, and power consumption of the refrigeration system. III-V compound semiconductor devices, although showing good response characteristics in the terahertz band, have a complex molecular beam epitaxy fabrication process, resulting in high unit costs and making it difficult to meet large-scale application requirements [10].

Perovskite materials have attracted considerable attention from researchers due to their advantages, such as high photoelectric conversion efficiency and tunable bandgaps. Early 3-D MAPbI_3_ exhibits excellent carrier transport and has advanced photovoltaics, yet its terahertz absorption is weak [11,12], and its environmental instability hampers large-scale use [13]. Two-dimensional perovskite PEA_2_PbI_4_ quantum wells shorten carrier relaxation to 20 ps, enabling 50 GHz THz modulation at 93% depth [14]. Halide-gradient “sheet engineering” further suppresses non-radiative recombination [15], while nonlinear THz fields directly tune Pb-halide octahedral vibrations to boost sensitivity [16]. Al_2_O_3_ passivation and MA^+^→FA^+^ cation engineering lock in stability. After 240 days in air, the imaging resolution is intact, and the room temperature *NEP* remains 5.03 pW Hz^−1^/^2^; accelerated aging under harsh T/H confirms reliability for real-world operation [17], ensuring stable performance in biologically relevant vapors and buffer salts. In parallel, Weyl semimetals have also attracted significant interest due to their high electron mobility and zero-bandgap characteristics. As a foundational benchmark established in 2018, the Weyl semimetal Co_3_Sn_2_S_2_ was demonstrated to exhibit ~2800 cm^2^ V^−1^ s^−1^ electron and ~2200 cm^2^ V^−1^ s^−1^ hole mobilities at 2 K despite ~10^19^ cm^−3^ carriers. This early milestone yielded a giant zero-field anomalous Hall conductivity of 1130 Ω^−1^ cm^−1^ and a record 20% Hall angle, laying the historical groundwork for low-dissipation, high-sensitivity topological spintronics [18].

Perovskite materials with high photoelectric conversion efficiency and Weyl semimetal materials with high carrier mobility both demonstrate significant potential in the field of terahertz detection devices. To overcome the intrinsic limitations of single-phase materials, constructing perovskite-based heterostructures has emerged as a highly effective strategy in recent optoelectronic research. The previous literature has extensively demonstrated that integrating perovskites with other functional materials—such as 2D transition metal dichalcogenides (TMDCs) or high-mobility inorganic semiconductors—can significantly facilitate ultrafast interfacial charge separation, passivate surface defects, and enhance environmental stability [19,20,21]. These heterojunction designs successfully mitigate non-radiative recombination and overcome the carrier extraction bottlenecks typically observed in pristine perovskite films. Inspired by these structural paradigms, this study fabricates a novel perovskite–Weyl semimetal heterostructure, which can effectively enhance carrier separation efficiency and mobility, thereby significantly improving the performance of terahertz detectors. Ultimately, the device achieved a responsivity of 374.15 A/W and a detectivity of 6.27 × 10^11^ Jones at room temperature, setting a new record for similar devices. These groundbreaking performance metrics—particularly its exceptional room temperature sensitivity, enhanced absorption across key spectral bands (including characteristic molecular fingerprint regions), and compatibility with silicon process integration—establish this detector as an ideal core component for constructing next-generation, highly integrated, low-cost, and portable terahertz spectroscopy and sensing systems.

## 2. Experimental Methods and Materials Design

### 2.1. Materials and Solution Preparation

The main materials used in the experiment include PbBr2 (>99.99%), CsBr (>99.9%), and PEACl (>99.5%), which were purchased from Xi’an Yuriguang Energy Technology Corporation (Xi’an, China). DABr (>99.5%) was purchased from J&K Scientific (Beijing, China), and DMSO (99.9%, anhydrous) was purchased from Sigma-Aldrich (Shanghai, China). All experimental materials were used directly after purchase without any purification. The perovskite precursor solution was prepared by mixing 42.56 mg of CsBr, 73.4 mg of PbBr_2_, 31.53 mg of PEACl, and 15.41 mg of DABr in 1 mL of DMSO solution. Before use, the solution was placed on a hot plate and heated at 45 °C for 30 min.

### 2.2. Device Fabrication and Film Preparation

The Co_3_Sn_2_S_2_ thin film is prepared by magnetron sputtering under optimized conditions to ensure uniformity, high quality, and minimal defects. The deposition process is carried out as follows. First, the chamber is evacuated to a base pressure of 1.0 × 10^−4^ Pa. Argon gas is then introduced into the cavity at a flow rate of 50 SCCM. Using an RF-powered magnetron source, a Co_3_Sn_2_S_2_ target is sputtered at a power of 120 W for a deposition time of 210 s. The substrate is mounted on a gold electrode with a gap distance of 5 mm. These parameters have been established through systematic experimentation to achieve reproducible and high-quality film growth.

The PEA-Cs_1−x_DA_x_PbBr_2.3_Cl_0.7_ perovskite film was prepared by the spin-coating method on the Co_3_Sn_2_S_2_ substrate. The prepared perovskite solution was coated on the Co_3_Sn_2_S_2_ substrate at a speed of 5000 revolutions per minute in a nitrogen-filled glove box for 60 s. The prepared perovskite film was then baked at 90 °C for 10 min.

### 2.3. Performance Testing and Characterization Methods

In this paper, the performance of terahertz detectors was systematically characterized by the bright-dark current contrast method with photocurrent excitation (including I-V curves, total noise Vn, responsivity *R_A_*, noise equivalent power *NEP*, detectivity D*, etc.). During the test, the device was placed at the center of the probe station, and two tungsten probes with a tip radius of curvature of 1 μm were used to form an ohmic contact with the metal electrodes. A measurement circuit was constructed by connecting a DC power supply (Keysight B2902A, Keysight Technologies, Santa Rosa, CA, USA) in series with a high-precision picoammeter (Keithley 6485, resolution 0.1 fA, Keithley Instruments, Solon, OH, USA). The terahertz signal was generated by an IMPATT avalanche diode source (TeraSense, center frequency 0.1 THz, maximum output power 70 mW, TeraSense Group, Inc., San Jose, CA, USA), and its output power was calibrated in real time by a terahertz power meter (ELVA-1 DPM, ELVA-1 Millimeter Wave Division, St. Petersburg, Russia). The effective detection area of the device was calibrated by a THz camera to be 1.2 mm^2^, which was significantly larger than the diffraction limit area at 0.1 THz frequency (about 0.03 mm^2^), thereby greatly improving the photon capture efficiency. To facilitate lateral comparison, the measured photocurrent data were converted to voltage responsivity based on the intrinsic resistance of the device (about 50 MΩ), a method that has been widely used in the performance evaluation of low-dimensional material terahertz detectors.

## 3. Results and Discussion

**Figure 1:**
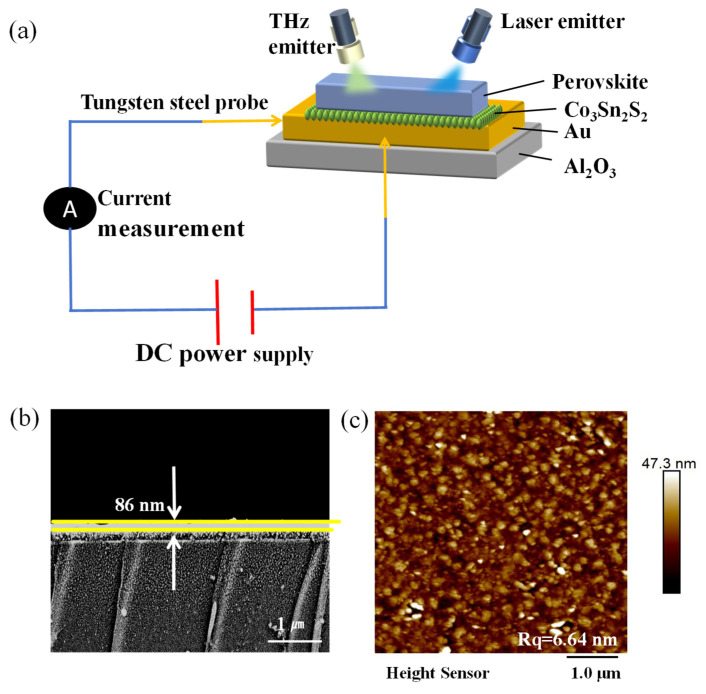
(**a**) Schematic diagram of the detector. (**b**) The thickness of the PEA-Cs_1−x_DA_x_PbBr_2.3_Cl_0.7_ film. (**c**) AFM image of the PEA-Cs_1−x_DA_x_PbBr_2.3_Cl_0.7_ film.

We fabricated PEA-Cs_1−x_DA_x_PbBr_2.3_Cl_0.7_ films by spin-coating, as shown in Figure 1b. The cross-sectional morphology of the PEA-Cs_1−x_DA_x_PbBr_2.3_Cl_0.7_ films was characterized by field emission scanning electron microscopy at an acceleration voltage of 5 kV in low current mode to minimize electron beam damage to the sample. The SEM images clearly revealed a distinct interface between the film and the substrate, with a smooth and uniform film surface. The average thickness of the film was measured at multiple points (*n* = 10) using ImageJ software (version 1.54r, National Institutes of Health, Bethesda, MD, USA), and the statistical results showed an average thickness of 86 ± 3.5 nm with a relative standard deviation (RSD) of 4.1%, indicating good thickness uniformity. This result was consistent with the thickness data obtained by ellipsometry (83 ± 5 nm), verifying the reliability of the measurement. The surface morphology of the spin-coated PEA-Cs_1−x_DAxPbBr_2.3_Cl_0.7_ films was analyzed by atomic force microscopy (AFM, Bruker Dimension Icon, tapping mode, RTESPA-300 probe, Bruker Corporation, Billerica, MA, USA). As shown in Figure 1c, the film surface presented a uniform distribution of nanocrystalline grains without obvious holes or cracks, indicating good film-forming properties of the precursor solution. Quantitative analysis using NanoScope Analysis software (version 2.0, Build R1Sr2.157217, Bruker Corporation, Billerica, MA, USA) revealed a root mean square roughness (Rq) of 6.64 nm for the film.

**Figure 2:**
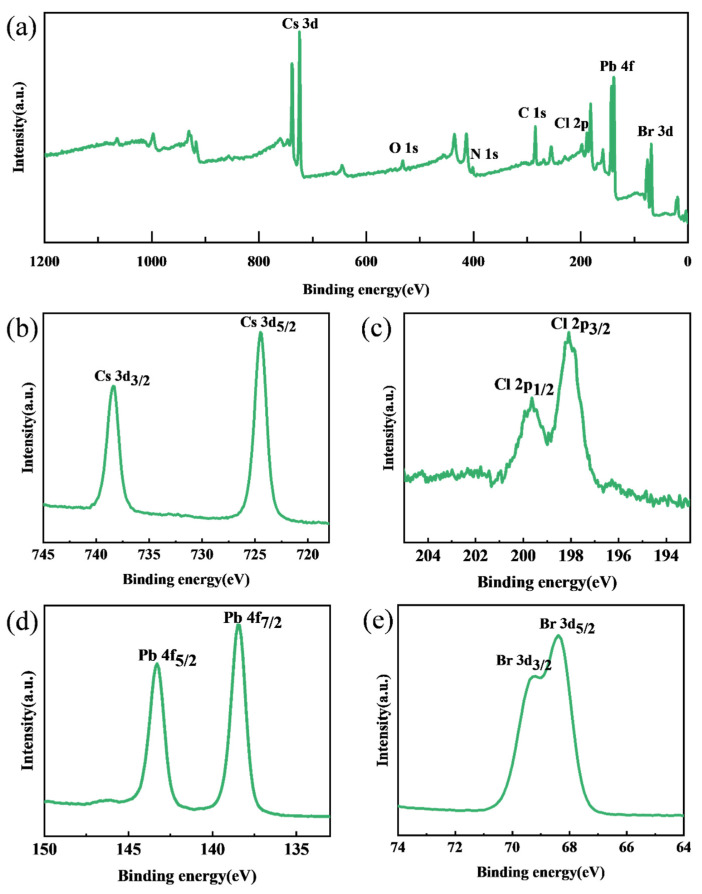
XPS spectra of the PEA-Cs_1−x_DA_x_PbBr_2.3_Cl_0.7_ perovskite films. (**a**) Full spectrum; (**b**) Cs 3d; (**c**) Cl 2p; (**d**) Pb 4f; (**e**) Br 3d.

The surface chemical states of PEA-Cs_1−x_DA_x_PbBr_2.3_Cl_0.7_ films were systematically characterized by X-ray photoelectron spectroscopy (XPS). As shown in Figure 2a, the full-spectrum scan revealed characteristic peaks of Br 3d, Pb 4f, Cl 2p, C 1s, N 1s, and Cs 3d on the sample surface, confirming the successful introduction of each constituent element in the perovskite film. High-resolution XPS spectra can provide detailed chemical state information of each element. Figure 2b shows the fine spectrum of the Cs 3d region, where two distinct peaks were observed at 724.5 eV and 738.4 eV, corresponding to the 3d_5/2_ and 3d_3/2_ spin-orbit split peaks of Cs^+^. This result indicates that the Cs element exists in the perovskite lattice in the +1 oxidation state, consistent with its theoretical valence. The high-resolution spectrum of Cl 2p (Figure 2c) shows two characteristic peaks at 198.1 eV and 199.7 eV, attributed to Cl 2p_3/2_ and Cl 2p_1/2_, respectively. The 1.6 eV energy level splitting is a typical feature of chlorine in perovskite structures, indicating that Br^−^ sites have been partially substituted by Cl^−^. The fine spectrum of Pb 4f (Figure 2d) presents two characteristic peaks at 138.5 eV (Pb^2+^ 4f_7/2_) and 143.3 eV (Pb^2+^ 4f_5/2_), with a spin-orbit splitting of 4.8 eV. No characteristic peaks of metallic Pb0 (usually appearing at lower binding energies) were observed in the spectrum, confirming the high phase purity of the perovskite film. The Br 3d spectrum (Figure 2e) shows characteristic peaks at 68.4 eV (Br 3d_5/2_) and 69.3 eV (Br 3d_3/2_), with a 0.9 eV energy level spacing consistent with reported values for bromine in perovskite materials. The symmetrical line shapes of these peaks further indicate that bromide ions are in a uniform chemical environment within the lattice. The comprehensive XPS analysis results demonstrate that the prepared PEA-Cs_1−x_DA_x_PbBr_2.3_Cl_0.7_ films have the expected chemical composition, and each element exhibits characteristic chemical states. No impurity phases or by-product signals were detected in the high-resolution spectra, confirming the high chemical purity of the samples.

**Figure 3:**
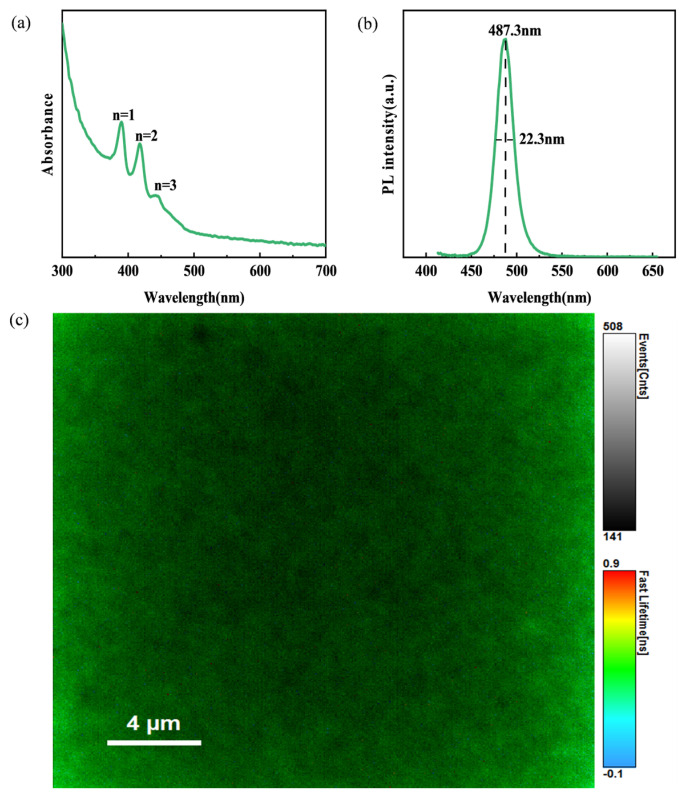
The optical properties of the PEA−Cs_1−x_DA_x_PbBr_2.3_Cl_0.7_ film. (**a**) Absorption spectra. (**b**) PL spectra. (**c**) PL mapping.

To comprehensively characterize the photophysical properties of the PEA-Cs_1−x_DA_x_PbBr_2.3_Cl_0.7_ perovskite films, we systematically conducted ultraviolet–visible absorption spectroscopy (UV-vis), steady-state photoluminescence (PL) spectroscopy, and micro-area photoluminescence imaging (PL mapping) tests. As shown in Figure 3a, the UV-vis absorption spectrum of the film exhibits typical perovskite material absorption features in the 300–700 nm wavelength range. Obvious absorption peaks were observed at 390 nm, 419 nm, and 445 nm, which correspond to the absorption edges of the pure 2D phase (*n* = 1), the 2D and 3D mixed phase (*n* = 2), and the pure 3D phase (*n* = 3), respectively. The absorption edge is located at approximately 510 nm, and the optical bandgap calculated from the Tauc plot is 2.43 eV, which is consistent with the reported bandgap value of PEA-based 2D perovskites. In the long-wavelength region (>500 nm), the absorption intensity gradually weakens but maintains a certain absorption tail, which may be due to the presence of localized states or defect states in the film. Steady-state PL spectra recorded under 405 nm excitation exhibit a narrow emission peak at 487 nm (2.54 eV) (Figure 3b), showing a Stokes shift of approximately 110 meV compared to the absorption edge, indicating a certain exciton–phonon coupling effect. The full width at half maximum (FWHM) of the emission peak is 22.3 nm, and the narrow peak width indicates good compositional uniformity of the film. To further study the uniformity of the film, we conducted PL mapping tests at a spatial resolution of 4 μm (Figure 3c). The relative standard deviation (RSD) of the PL intensity over the entire scanning area is 8.7%, indicating good uniformity of the film. It was observed that the PL intensity in the edge region is slightly lower than that in the central region, which may be due to the edge effect generated during the spin-coating process. The PL peak position mapping shows that the emission wavelength variation range across the entire area is less than 3 nm.

**Figure 4:**
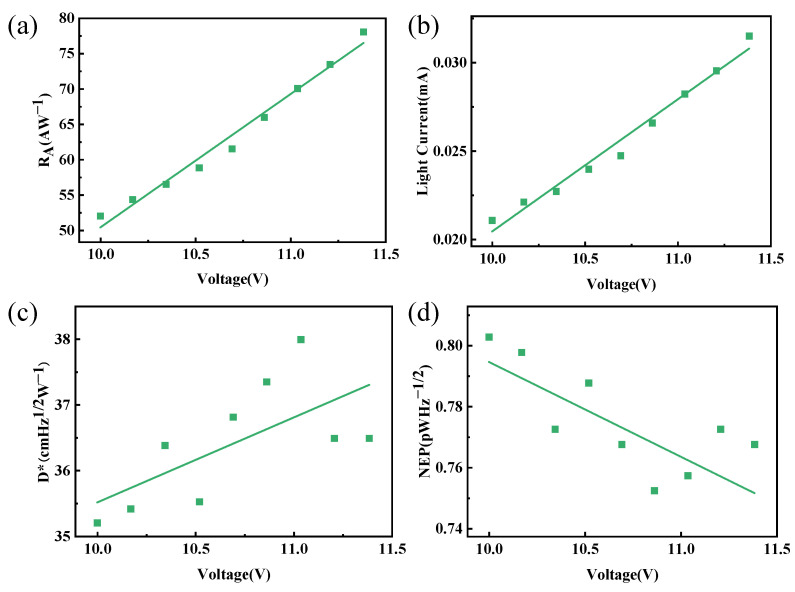
The performance of the independent Co_3_Sn_2_S_2_ device: (**a**) Light current, (**b**) *R_A_*, (**c**) D*, and (**d**) *NEP*.

To explicitly elucidate the active contribution of the perovskite layer, a standalone Co_3_Sn_2_S_2_ device was fabricated and characterized as a control. As depicted in Figure 4, the standalone Weyl semimetal device exhibits a limited THz response, with an *R_A_* of only 78.09 A/W, an *NEP* of 0.77 pW·Hz^−1/2^, and a D* of 38.0 cm Hz^1/2^·W^−1^. In stark contrast, the perovskite/Co_3_Sn_2_S_2_ heterojunction achieves a dramatically enhanced *R_A_* of 338.58 A/W, along with a reduced *NEP* of 0.34 pW·Hz^−1/2^ and an improved D* of 54.43 × 10^10^ cm·Hz^1/2^·W^−1^. This massive performance gap confirms that the perovskite film does not act merely as a passive capping layer. Instead, it plays a vital active role by efficiently absorbing incident photons and generating carriers that are subsequently injected into the Co_3_Sn_2_S_2_ channel. This direct comparative analysis unambiguously substantiates that the high performance originates from the strong synergistic effect between the two materials.

**Figure 5:**
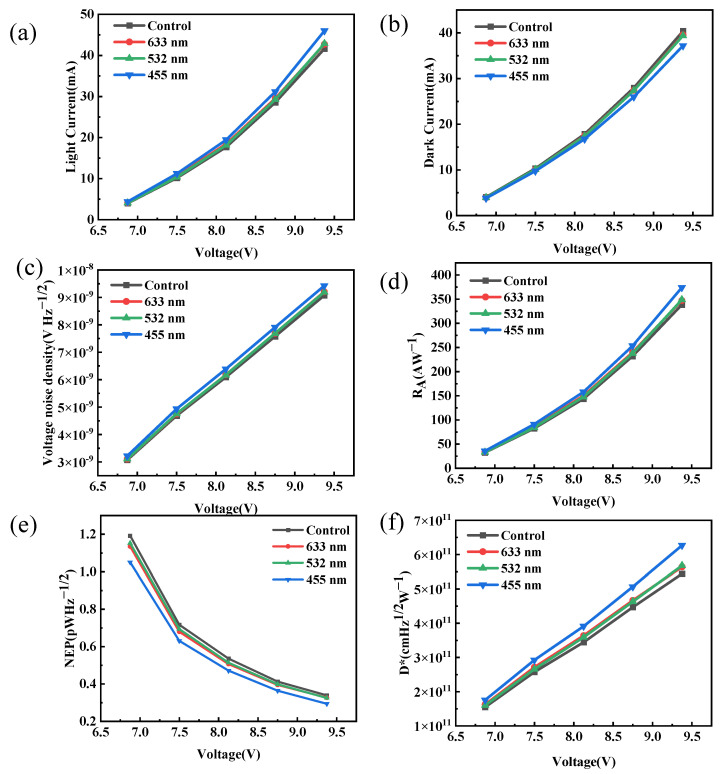
(**a**) Light current, (**b**) dark current with the applied voltage, (**c**) total noise, (**d**) *R_A_*, (**e**) *NEP*, and (**f**) D* of the devices at different voltage biases for room temperature operation at 0.1 THz.

Figure 5 shows the photocurrent, dark current, and noise characteristics. The test results indicate that the device exhibits excellent ohmic contact characteristics, which guarantee efficient carrier transport. By comparing the performance under different wavelength laser irradiation, we find that, as shown in Figure 5a,b, the photocurrent and dark current levels of the device under 633 nm and 532 nm laser irradiation exhibit no significant differences when compared to the untreated reference sample. In contrast, 455 nm laser irradiation leads to clear performance variations. As detailed in Table 1, the absolute photocurrent increases by 10.5% (from 0.0416349 mA to 0.0460088 mA), while the dark current is reduced by 8.0% (from 0.0404009 mA to 0.0371763 mA) relative to the reference device. The performance enhancement under 455 nm laser irradiation is attributed to a photogating effect. Since the photon energy (2.73 eV) exceeds the perovskite bandgap (2.43 eV), standard inter-band absorption occurs. This generates a steady-state population of trapped carriers that shifts the quasi-Fermi level, thereby modulating the channel conductance and boosting the device responsivity. While this pronounced photogating effect clearly indicates effective carrier manipulation, the precise charge transfer mechanism at the perovskite/electrode interface warrants detailed discussion. Drawing insights from recent fundamental studies on perovskite heterostructures [22], the efficient charge extraction is largely driven by the favorable energy band alignment and strong interfacial coupling. Upon illumination, the photogenerated electron–hole pairs in the perovskite undergo rapid dissociation. The built-in potential at the interface facilitates the swift transfer of carriers into the adjacent functional layer while suppressing non-radiative recombination. Although future time-resolved photoluminescence (TRPL) studies are required to experimentally quantify these ultrafast extraction dynamics for this specific device, the robust macroscopic optoelectronic performance strongly corroborates this highly efficient charge transfer pathway. In contrast, the photon energies of 633 nm (1.96 eV) and 532 nm (2.33 eV) deviate from the optimal absorption range. To further identify the noise, the noise current of the photocurrent at 0.1 THz was analyzed using the Fourier Transform (FFT). As shown in Figure 5c, the noise voltages of both the experimental group device and the reference device show a clear upward trend, and the four curves have similar trends and almost overlap. Notably, the noise voltage of the 455 nm device is only 3.8% higher than that of the reference device (at 9.4 V), indicating that the influence of different wavelength treatments on the noise characteristics is limited. The photoelectric conversion capability of the detector is evaluated by its responsivity (*R_A_*), noise equivalent power (*NEP*), and detectivity (D*). These performance indicators of *R_A_*, *NEP*, and D* can be expressed by the following formula [23,24]:

(1) $$R_{A}=\frac{I}{P}$$

(2) $$NEP=\frac{V_{n}}{R_{A}}$$

(3) $$D^{*}=\frac{\sqrt{S}}{NEP}$$

I represents the photocurrent of the device, P represents the incident terahertz power, S represents the effective detection area of the detector, and Vn is the noise voltage.

At a room temperature of 0.1 terahertz, the *R_A_* of the device under different voltage biases is shown in Figure 5d. As comprehensively summarized in Table 1, when the voltage is 9.4 V, the *R_A_* of the 455 nm laser-treated sample reaches 374.15 A/W, exhibiting a 10.5% improvement compared to the reference sample (338.58 A/W). In Figure 5e, we can observe that the *NEP* shows a typical voltage dependence and tends to saturate when the voltage is greater than 10 V. At 9.4 V, the *NEP* of the 455 nm laser-treated sample is as low as 0.29 pW/Hz^1/2^. This is two orders of magnitude better than commercial pyroelectric detectors (~50 pW/Hz^1/2^). D* (Figure 5f) increases with the increase in voltage. Based on the exact values provided in Table 1, the D* of the experimental group under 455 nm laser irradiation reaches 6.27 × 10^11^ cm Hz^1/2^ W^−1^ at 9.4 V, which is 15.3% higher than that of the reference sample (5.44 × 10^11^ cm Hz^1/2^ W^−1^). This sets a performance record for room temperature detectors in the same frequency band.

## 4. Conclusions

This study successfully developed a high-performance terahertz detector based on perovskite materials. The device achieved a high *R_A_* of 374.15 A/W, an extremely low *NEP* of 0.29 pW/Hz^1/2^, and D* reaching 6.27 × 10^11^ cm Hz^1/2^ W^−1^ under a 9.4 V bias. Notably, this extremely low *NEP* represents a nearly 500-fold improvement over commercial Golay detectors (~1.4 × 10^−10^ W/Hz^1/2^). Furthermore, the photovoltaic response was significantly enhanced under 455 nm laser ir_ra_diation. Through innovative band engineering and interface control, we demonstrate that this enhancement is driven by a pronounced photogating effect, which shifts the Fermi level and boosts detection sensitivity while maintaining high response speeds. While this detector exhibits exceptional intrinsic room temperature sensitivity and potential for low-cost manufacturing, the unencapsulated perovskite active layer remains sensitive to ambient conditions. Therefore, this work primarily serves as a vital proof-of-concept for these new optoelectronic mechanisms. Future research will focus on implementing robust encapsulation strategies to translate these high-performance, cost-efficient characteristics into practical, long-term solutions for advanced material sensing, 6G communications, and security detection.

## Figures and Tables

**Table 1:** The performance of the detector under different wavelength processing conditions.

	Light Current (mA)	Dark Current (mA)	*Vn* (10^−9^ V·Hz^−1/2^)	*R_A_* (A/W)	*NEP* (pW/Hz^1/2^)	D* (10^10^ cm·Hz^1/2^·W^−1^)
Control	0.0416349	0.0404009	9.08	338.58	0.34	54.43
633 nm	0.0427139	0.0395534	9.21	347.36	0.33	56.44
532 nm	0.0428842	0.0393797	9.19	348.74	0.32	56.78
455 nm	0.0460088	0.0371763	9.43	374.15	0.29	62.70

## Data Availability

The original contributions presented in this study are included in the article. Further inquiries can be directed to the corresponding authors.
